# Comparative Effectiveness of Epidural Steroid İnjections in Patients With Disc Bulging and Disc Protrusion

**DOI:** 10.7759/cureus.45994

**Published:** 2023-09-26

**Authors:** Eyup Çetin, Volkan Şah, Irfan Zengin, Özkan Arabacı, Mehmet E Akyol, Murat Yücel

**Affiliations:** 1 Department of Neurosurgery, Haydarpaşa Numune Education and Research Hospital, Istanbul, TUR; 2 Department of Sports Medicine, Van Yuzuncu Yıl University, Van, TUR; 3 Department of Neurosurgery, Faculty of Medicine, Yuzuncu Yil University, Van, TUR; 4 Department of Neurosurgery, Van Yuzuncu Yıl University, Van, TUR; 5 Department of Neurosurgery, Yalova University, Yalova, TUR

**Keywords:** odi score, vas score, discogenic low back pain, protrusion, bulging, epidural steroid

## Abstract

Purpose

Discogenic low back pain is a widespread disorder in the world. Many methods have been developed and continue to be developed in the treatment of discogenic low back pain. We aimed to examine the effect of epidural steroid administration on disc bulging and disc protrusion in patients with discogenic low back pain.

Design

The clinical effects of interlaminar epidural steroids administered to 71 patients who were admitted to our clinic and whose treatment did not require surgery were radiologically divided into two groups disc bulging and disc protrusion. Patients were followed up for six months and clinical results were recorded.

Methods

The scores of the Visual Analog Scale (VAS) and Oswestry Disability Index (ODI) were measured before the procedure, one week after the procedure, one month after the procedure, and six months after the procedure. The normal distribution of continuous variables was evaluated using the Kolmogorov-Smirnov test. Continuous variables were compared with the Mann-Whitney U test and categorical variables were compared using the Chi-square test or Fisher's exact test.

Results

There was no significant difference in demographic data in patients with disc bulging and disc protrusion. In the disc protrusion group, the VAS scores in the first week after, the first month after and the sixth month after the procedure showed a significant decrease compared to the pre-procedure. There was no significant difference between the disc bulging and protrusion groups in the first week of post-procedure VAS score reduction. In the Disc Bulging group, the ODI score one week after, one month after, and six months after the procedure showed a significant decrease compared to the pre-procedure. In the Disc Protrusion group, the ODI score one week after, one month after, and six months after the procedure showed a significant decrease compared to the pre-procedure.

Conclusion

There was strong evidence that lumbar interlaminar steroid injection is an effective treatment for disc bulging and discogenic pain due to protrusion. When the ODI and VAS scores of patients with both disc bulging and disc protrusion were evaluated, it was seen that they benefited from epidural steroid injection. In the disc protrusion group, except for the first week of injection, relief due to the use of epidural steroids was observed to be greater compared to disc bulging.

## Introduction

Low back pain is one of the leading causes of decreased quality of life [1]. It is also a major cause of physical disability, affecting both older and younger people, and can have enormous socioeconomic and health implications [2]. Low back pain usually resolves in three months; if it does not improve within more than three months, it is considered chronic [3].

One of the main causes is aging-related intervertebral disc degeneration, which affects the nervous system around the disc [4]. “Discogenic pain” occurs with the stimulation of nociceptors in the annulus fibrosus [5]. Degeneration stimulates pain receptors in the disc with endplate damage and inflammation, however, it can leave the outer disc intact [6]. Disc degeneration can be defined as the process involving changes in the tissue and cellular microenvironment, eventually leading to structural deterioration and disruption of the intervertebral disc [7]. The presence of disc tissue extending beyond the edges of the ring apophyses, throughout the circumference of the disc, is termed “bulging” and is not considered a form of herniation. Disc protrusion is present if the greatest distance between the edges of the disc material presenting outside the disc space is less than the distance between the edges of the base of that disc material extending outside the disc space [8].

A number of methods are used for the treatment of discogenic low back pain [9]. Treatments for discogenic pain often focus on surgical procedures such as fusion and total disc replacement. The safety and efficacy of these surgical procedures are still controversial, as it has been reported to reduce only the pain [10]. Conservative treatments such as bed rest, physical therapy, medication, or injections have been used to treat discogenic pain [11]. Previously reported clinical data also mention the efficacy and feasibility of biomolecular and cellular therapies for the treatment of degenerative disc disease [12].

The use of non-surgical interventions, including injection therapy, is increasing rapidly and steroids and/or local anesthetics are predominantly used in injections. Epidural steroid injection (ESI) is widely used for the treatment of low back pain, despite the controversy regarding the appropriate use of injection therapies [13]. ESI treatment primarily aims to relieve pain. However, allowing a patient to progress in an exercise and rehabilitation program can also be considered an advantage, thus contributing to the patient's long-term outcome [14]. In the USA, approximately nine million ESI are administered annually to treat radicular back pain [15]. This form of treatment usually provides pain relief and functional improvement for patients and also does not have serious side effects [16]. The concern for elderly patients is that ESI poses a potential risk of osteoporosis [17].

Systemic exposure to oral and intravenous glucocorticoids is much higher than with ESIs. Oral and intravenous glucocorticoids have profound detrimental effects on bone and rapidly increase the risk of fracture in the first three months of use [18]. There are two types of ESI preparations: particulated and non-particulated. Triamcinolone, betamethasone, and methylprednisolone are particulated glucocorticoids, while dexamethasone is a non-particulated glucocorticoid. Particulate glucocorticoids have a lower solubility and therefore a longer duration of action. However, they generally tend to have a higher risk of embolic events [19]. This resulted in an FDA warning regarding their use; however, it is still frequently used as some studies report better short-term results with these preparations [20].

## Materials and methods

This study was conducted in a prospective observational study using the data of the patients who had regular follow-up from the records of the patients who underwent ESSI in our clinic between June 2021 and June 2022 at Van Yüzüncü Yıl University Dursun Odabaşı Hospital. Written and verbal information was given to all patients participating in the study. Ethics committee approval was received from Van Yüzüncü Yıl University Ethics Committee with the decision letter number 7 on December 25, 2020 and a permission letter from Van Yüzüncü Yıl University Dursun Odabaşı Hospital management. This article was previously posted to the Research Square preprint server on January 11, 2023.

The purpose of the study was explained to all patients and their written consent was obtained. Our study was conducted on the records of a total of 71 patients with discogenic low back pain and radiologically detected disc bulging or disc protrusion. The Visual Analog Scale (VAS) and Oswestry Disability Index (ODI) scores of the patients before the procedure, one week after the procedure, one month after the procedure, and six months after the procedure were recorded, and the responses obtained from the treatment of the patients were recorded as measurements.

Demographic characteristics of patients (age, gender), comorbidities, height, weight, body mass index, duration of complaint (less than three months, between three and six months, longer than six months), VAS score, ODI score, disc level, clinical findings, disc radiology was recorded.

Intervention

After determining the level of lumbar disc pathology, the operation area was wiped three times with an antiseptic solution containing batikon. After providing local anesthesia with bupivacaine, which was prepared previously under sterile conditions, the epidural space was reached with an interlaminar approach with a 17-gauge spinal needle. At the same time, loss of resistance was observed with the injector filled with bupivacaine through the spinal needle. At the point of loss of resistance, a total of 8cc mixture (two ampoules of depot steroid containing 40 mg of methylprednisolone acetate in 1 mL and 6 cc of saline) was injected into the epidural space. Afterward, the patient's neurological control was ensured, and she/he was discharged with recommendations.

Visual Analog Scale

It is a scale consisting of a single line of 10 cm and evaluating the severity of pain. The patients were asked to mark the severity of their pain at rest and during activity on two separate 10 cm lines. The patients were given the starting point of the scale as no pain. The last point was expressed as the most severe pain encountered in life. During the calculation, the distance between the marked point and the starting point is measured in cm. An increase in the score means that the severity of pain increases.

Oswestry Disability Index

It was developed to evaluate the degree of loss of function in low back pain and consists of 10 items. The items question the severity of pain, self-care, lifting-carrying, walking, sitting, standing, sleep, the degree of change in pain, travel, and social life. Under each item, there are six statements that the patient marked as appropriate for his/her condition. The first statement is scored as “0” and the sixth statement is scored as “5.” When the total score is calculated, it is multiplied by two and expressed as a percentage. The maximum score is "100," the minimum score is "0." Toplam skor arttıkça özürlülük düzeyi de artmaktadır.

Statistical method

Mean, standard deviation, median minimum, maximum, frequency, and ratio values were used in the descriptive statistics of the data. The distribution of variables is measured with the Kolmogorov-Smirnov test. Independent sample t-test and Mann-Whitney U test were used in the analysis of quantitative independent data. Wilcoxon test was used in the analysis of dependent quantitative data. The chi-square test was used in the analysis of qualitative independent data, and the Fischer test was used when the chi-square test conditions were not met. SPSS 28.0 (IBM Corp., Armonk, NY) program was used in the analysis.

## Results

Age and gender distribution of the patients did not differ significantly between the Disc Bulging and Protrusion groups (p > 0.05). Height, weight and BMI values did not differ significantly (p > 0.05) between Disc Bulging and Protrusion groups. The pain duration did not differ significantly (p > 0.05) between the Disc Bulging and Protrusion groups. The clinical status characteristics did not differ significantly (p > 0.05) between the Disc Bulging and Protrusion groups. The distribution of co-morbid disease rates between the Disc Bulging and Protrusion groups did not differ significantly (p > 0.05) (Table 1).

**Table 1:** Demographic data of disc patients t - Independent sample t-test, m - Mann-Whitney U test, X² - Chi-square test

	Disc Bulging					Disc Protrusion				p	
		Mean±sd/n-%			Median		Mean±sd/n-%					Median
Age		41.3	±	14.0	40.5		46.8	±	12.9	45.0	0.060	^m^
Gender	Female	13		38.2%			21		56.8%		0.119	^X²^
	Male	21		61.8%			16		43.2%			
Height		169.8	±	8.8	170.0		167.2	±	7.4	165.0	0.190	^t^
Weight		77.8	±	9.9	76.0		76.9	±	12.1	76.0	0.740	^t^
BodyMassIndex		27.0	±	3.1	26.7		27.5	±	4.2	27.3	0.569	^t^
Pain Duration	<3 Months	16		47.1%			15		40.5%		0.672	^X²^
	3-6 Months	7		20.6%			11		29.7%			
	> 6 Months	11		32.4%			11		29.7%			
Physical Examination	Normal	14		41.2%			9		24.3%		0.130	^X²^
	Laseque Pozitive	20		58.8%			22		59.5%		0.957	^X²^
	Sensory Deficit	0		0.0%			3		8.1%		0.241	^X²^
	Motor Deficit	0		0.0%			3		8.1%		0.241	^X²^
Comorbidity	Not Exist	27		79.4%			27		73.0%		0.525	^X²^

There was no significant difference (p > 0.05) in the pre-procedure and post-procedure first week VAS scores between the Disc Bulging and Protrusion groups. In the Disk Bulging group, the VAS score at week 1, one month after the procedure, and six months after the procedure decreased significantly (p < 0.05) compared to the pre-procedure. In the Disc Protrusion group, the VAS score at week 1, one month after the procedure, and six months after the procedure decreased significantly (p < 0.05) compared to the pre-procedure. There was no significant difference (p > 0.05) in the first week post-procedure VAS score reduction between the Disc Bulging and Protrusion groups. In the Disk Protrusion group, the first month and the sixth month post-procedure VAS score decrease was significantly higher (p < 0.05) than the Disk Bulging group (Table 2).

**Table 2:** Comparison of disc bulging and disc protrusion results m - Mann-Whitney U test, w - Wilcoxon test

	Disc Bulging					Disc Protrusion				p	
		Mean ±sd			Median		Mean ±sd					Median
VAS Scores												
Baseline		7.1	±	1.2	7.0		7.4	±	1.3	8.0	0.432	^m^
1th week of ESI		0.8	±	1.0	0.5		0.6	±	0.8	0.0	0.533	^m^
1th month of ESI		3.3	±	2.0	4.0		2.3	±	1.5	2.0	0.017	^m^
6th month of ESI		1.5	±	1.4	1.5		0.9	±	1.3	0.0	0.023	^m^
Difference between pre and post ESI												
Baseline-1th week		-6.3	±	1.4	-6.0		-6.7	±	1.6	-7.0	0.228	^m^
Intra-Group change p		0.000			^W^		0.000			^W^		
Baseline-1th month		-3.9	±	2.5	-4.0		-5.1	±	1.8	-5.0	0.028	^m^
Intra-Group change p		0.000			^W^		0.000			^W^		
Baseline-6th month		-5.6	±	1.8	-5.5		-6.5	±	1.7	-7.0	0.048	^m^
Intra-Group change p		0.000			^W^		0.000			^W^		
ODI Scores												
Baseline		67.0	±	8.3	66.0		68.0	±	8.6	68.0	0.446	^m^
1th week of ESI		15.5	±	11.4	14.0		13.1	±	10.0	12.0	0.354	^m^
1th month of ESI		38.9	±	18.8	37.0		31.7	±	13.8	32.0	0.100	^m^
6th month of ESI		19.2	±	15.3	14.0		14.6	±	13.1	12.0	0.187	^m^
Difference between pre and post ESI												
Baseline-1th week		-51.5	±	13.6	-50.0		-54.9	±	14.8	-54.0	0.261	^m^
Intra-Group change p		0.000			^W^		0.000			^W^		
Baseline-1th month		-28.1	±	21.2	-28.0		-36.3	±	14.7	-34.0	0.092	^m^
Intra-Group change p		0.000			^W^		0.000			^W^		
Baseline-6th month		-47.8	±	18.5	-50.0		-53.4	±	14.5	-54.0	0.307	^m^
Intra-Group change p		0.000			^W^		0.000			^W^		

There was no significant difference (p > 0.05) in the ODI score before the procedure, at the first week after the procedure, at the first month after the procedure, and at the sixth month after the procedure between the Disc Bulging and Protrusion groups. In the Disk Bulging group, the ODI score at one week, one month after the procedure, and six months after the procedure decreased significantly (p < 0.05) compared to the pre-procedure. In the Disc Protrusion group, the ODI score at one week, one month after the procedure, and six months after the procedure decreased significantly (p < 0.05) compared to the pre-procedure. The amount of ODI score decrease at the first week, first month after the procedure, and sixth month after the procedure did not differ significantly between the Disc Bulging and Protrusion groups (p > 0.05) (Table 2).

## Discussion

The mechanism of ESI-induced pain relief is thought to be multifactorial. Corticosteroids inhibit phospholipase A2, which converts membrane phospholipids to arachidonic acid and lysophospholipids [21]. Arachidonic acid is then converted to proinflammatory eicosanoids, including prostaglandins, prostacyclins, thromboxanes, and leukotrienes. These inflammatory mediators can exacerbate pain and sensitize peripheral nociceptors. In addition to their anti-inflammatory effects, corticosteroids can inhibit ectopic discharges from nerve fibers and suppress the conduction of unmyelinated fibers [22].

In some cases, the inflammatory process associated with disc herniation may not be alleviated by non-steroidal anti-inflammatory drugs or oral steroids but can be reduced by ESI. Although the reason for this observation is not fully known, we hypothesize that it may be related to repetitive systemic steroid uptake from the epidural veins in the posterior epidural space and also from the blood vessels in the subarachnoid space after passive diffusion of steroids through the dura [23]. ESIs, which are widely used in the treatment of low back and lumbosacral radicular pain, can be performed with interlaminar, caudal, and transforaminal (TF) approaches [24]. There have been studies showing that the translaminar and TF approaches are more effective than the caudal approach in patients with disc herniation [25]. The interlaminar approach is widely used, but debate continues about its effectiveness. The possible reason for this may be the limited ventral epidural spread due to the fact that the drug is mainly administered to the dorsal epidural space [26]. Recently, an ESI with an oblique interlaminar approach has been described. It was stated that this approach is equivalent to the TF approach in pain relief and functional improvement in the treatment of low back and unilateral lumbosacral radicular pain [27].

Several studies have shown that an ESI delivered via the parasagittal interlaminar approach is equivalent to providing pain relief and functional improvement via the TF approach for the treatment of lower back and lumbosacral radicular pain [28]. The effectiveness of ESI treatment depends on whether the steroids reach the nerve root lesion. Most disc herniations or nerve root compression lesions are located at the back of the vertebra; therefore, it is suggested that an ESI will be more effective if delivered close to this targeted site [29].

Epidural injections may work in part by lavage of the epidural space or possibly by breaking up epidural and nerve root adhesions. In a systematic review, Rabinovitch et al. found a strong correlation between epidural injection volume and outcome, regardless of steroid dose [30].

Complications in lumbar ESIs are extremely rare and most of them can be prevented with correct needle placement, sterile techniques, and fluoroscopically guided injections [31]. In our study, no serious complication was observed in either group; but one patient had severe headache. Acute chemical meningitis may rarely develop after ESI [32].

In a retrospective study by You-Ri et al., clinical similarities and differences in acute bacterial and chemical meningitis were revealed. In their study, it was shown that C-reactive protein (CRP) and procalcitonin were significantly lower than that of meningitis after ES, with the clinical findings being similar [33]. Since the follow-up period of our study was not long, we could not evaluate the long-term side effects of corticosteroid use. In studies conducted between 1997 and 2014, in terms of serious neurological complications associated with ESIs; a total of 90 serious and sometimes fatal neurological events were reported, including cases of paraplegia, quadriplegia, spinal cord infarction, and stroke [34]. In general, sciatic pain from a lumbar disc herniation is a self-limiting condition that resolves within weeks or months without medical attention; In some cases, rest, analgesic medications, and a structured exercise program may be required. Unless there is a neurological deficit, the inflammatory state is generally more important than mechanical compression in the pathogenesis and chronicity of the disease. However, surgery is generally recommended for patients who are resistant to conservative treatment [35].

ESI is often used for the treatment of lumbosacral radiculopathy and is recommended in various guidelines and by multiple pain societies [28]. In studies comparing the pulsed radiofrequency method, which has been used widespread recently, and ESI, studies have been published showing that although the radiofrequency method is superior in the short term, ESI is superior in the long term [36]. In fact, there are reports that it is necessary to repeat steroid injections in symptomatic patients with lumbar disc herniation whose symptoms do not improve satisfactorily after the first injection [37].

Although there are many studies in the literature on the long and short-term effects of epidural steroids on patients with disc herniation and spinal stenosis, there is no comparative study on disc bulging and disc protrusion. In our study, we examined the efficacy of epidural steroid administration on patients with discogenic low back pain who showed characteristic disc bulging or disc protrusion on radiological lumbar imaging.

The efficacy of ESI is controversial given the lack of control and quality of most studies. In our study, it was observed that epidural steroid administration relieved discogenic low back pain similar to the literature [38]. The reason for the different results in the literature is thought to be the effect of steroid type, frequency of administration, injection volume, radiological characteristics of the disc, and the absence of precise guides. In addition, the fact that most of these studies were performed without fluoroscopic control is an important reason that changes the results.

In our study, there was no significant difference in terms of ODI score in the disc protrusion and disc bulging groups in the pre- and post-procedure follow-ups. But VAS score was significantly lower in the protrusion group only in the first month after the procedure and in the sixth month after the procedure. In our study, it was observed that the use of epidural steroids had positive effects on patients in the short and long term, with similar efficacy in both groups. Overall, according to a consistent number of studies and the guidelines written by Kreiner et al., ESIs appear to be safe and highly effective in relieving major symptoms and delaying surgery, especially in short-term follow-ups [39].

**Figure 1:**
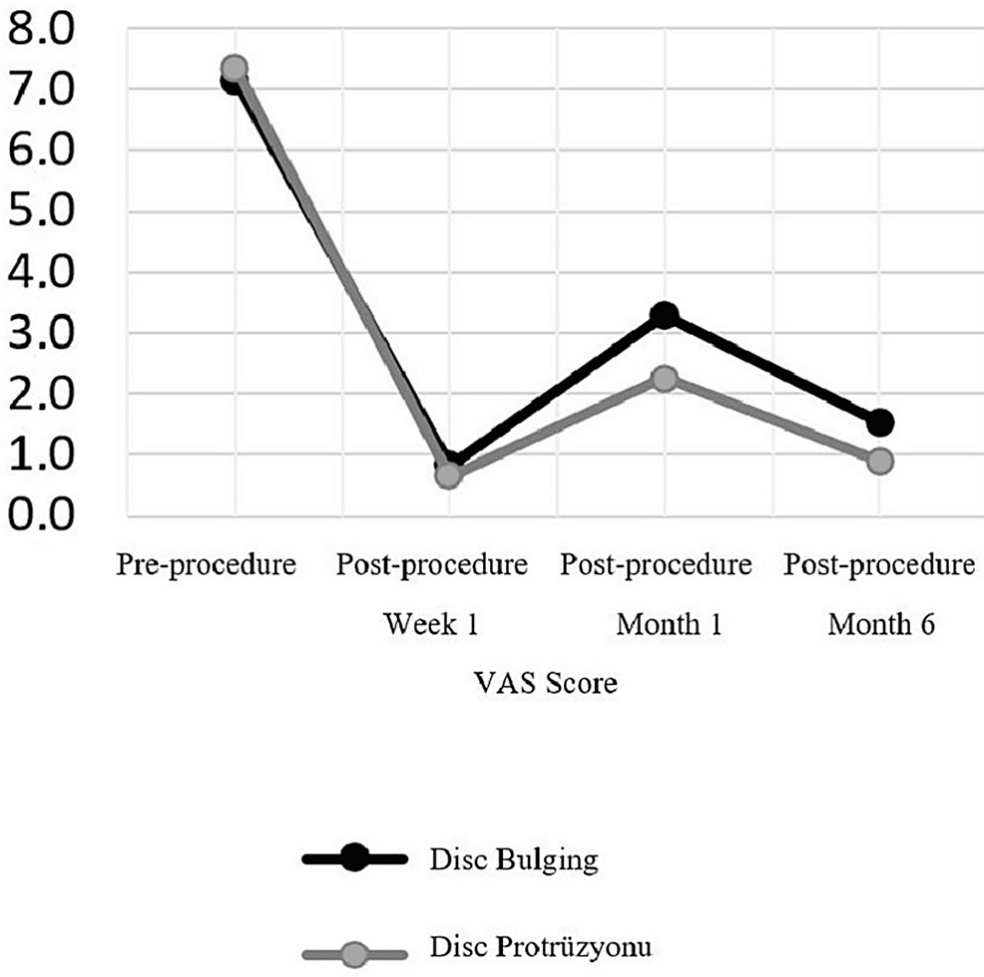
VAS scores according to evaluation periods VAS - Visual Analog Scale

**Figure 2:**
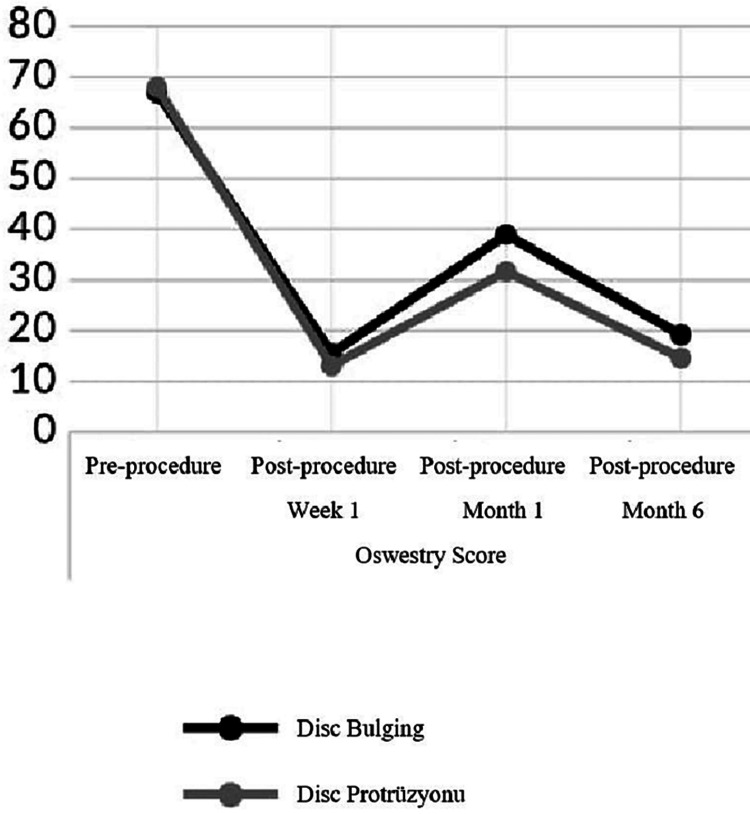
ODI scores according to evaluation periods ODI - Oswestry Disability Index

Although there is a decrease in the efficacy of the drug in the first month after epidural steroid administration with both VAS score and ODI score in both groups, it is seen that the degree of effectiveness is higher than before the procedure. In the literature, studies comparing spinal stenosis and disc herniation have shown that ESI therapy is more effective in disc herniation [40]. Our study will contribute to the literature as the first publication examining the efficacy of epidural steroid administration by distinguishing the disc as bulging and protrusion radiologically (Figures 1, 2).

## Conclusions

According to our study, it is observed that epidural steroid administration is still an effective and safe method in the treatment of discogenic pain and it showed similar positive results on both disc bulging and disc protrusion. While ESIs appear to be effective in relieving symptoms and delaying surgery in the short term, evidence of long-term benefits is still lacking. More studies are needed to better understand which patients might benefit more from ESIs.
